# Supplementary material for: Knowledge of Community Pharmacists in Saudi Arabia Regarding Human Monkeypox, Its Management, Prevention, and Vaccination: Findings and Implications
Source: Vaccines (Basel). 2023 Apr 21;11(4):878. doi: 10.3390/vaccines11040878 (PMC10143221; doi:10.3390/vaccines11040878)
Supplement: Supplementary file 1 [file vaccines-11-00878-s001.zip › vaccines-2324222-supplementary.pdf]

Supplementary Tables

Table S1: Survey of Knowledge of community pharmacists regarding human monkeypox

Demographic data:

1. Gender

- ☐ Male
- ☐ Female

2. Age

- ☐ 24-30
- ☐ 31-40
- ☐ 41-50
- ☐ >50

3. Qualifications

- ☐ BPharm
- ☐ PharmD
- ☐ Postgraduate Diploma
- ☐ MSc/MPharm/equivalent or higher

4. Experience as a community pharmacist in Saudi Arabia (in years)

- ☐ Less than 1 year
- ☐ 1 to 5 years
- ☐ 6 to 10 years
- ☐ More than 10 years

Knowledge statements:

No.	Statement	Yes	No	I do not know
1	Monkeypox is caused by a virus (i.e. a viral disease infection)	◆		
2	Monkeypox is a newly discovered disease in humans in 2022		◆	
3	Globally, in this year 2022, monkeypox has caused less than 10000 cases till now		◆	
4	In Saudi Arabia, there are reported cases of monkeypox in 2022	◆		
5	The current global outbreak of monkeypox is declared by the World Health Organization (WHO) a Public Health Emergency of International Concern	◆		
6	Skin rashes and lesions are one of the key characteristics for identifying monkeypox	◆		
7	The following are signs and symptoms that can occur in persons with monkeypox:			
	Fever	◆		
	Chills	◆		
	Lymphadenopathy (swelling of the lymph nodes)	◆		
	Headache	◆		
	Exhaustion/lack of energy	◆		
	Muscle aches	◆		
	back pain	◆		
	Respiratory symptoms (e.g. sore throat, nasal congestion, cough)	◆		
8	The incubation period of monkeypox (i.e., time from exposure to the pathogen to onset of symptoms) can range from 1-5 days		◆	
9	Monkeypox symptoms typically last from 2 to 4 weeks	◆		
10	The case-fatality rate (معدل الوفاة) for monkeypox infection has been estimated at approximately 30%		◆	
11	Human-to-human transmission can occur via close contact with skin lesions or respiratory secretions of an infected person with monkeypox	◆		
12	Human-to-human transmission can occur via prolonged face-to-face contact with an infected person	◆		
13	Persons could get infected via direct contact with objects, surfaces, or materials contaminated with the monkeypox	◆		
14	Animal-to-human transmission can occur via eating inadequately cooked meat and other animal products of an infected animal	◆		
15	Asymptomatic individuals (i.e. during the incubation period of the infection) are the main source of spreading the monkeypox infection		◆	
16	Diagnosis of monkeypox infection is confirmed by using a real-time polymerase chain reaction (PCR) test	◆		
17	Medications such as tecovirimat and brincidofovir that were developed to treat smallpox may be used to treat monkeypox	◆		
18	There are approved vaccines for the prevention of monkeypox	◆		
19	Standard preventive and precautionary measures are effective in preventing monkeypox	◆		
20	Supportive care and adequate hydration may be enough for most patients with an intact (healthy) immune system	◆		
21	Patients with severe monkeypox symptoms or at increased risk to develop severe monkeypox infection should be referred for a specialized medical care	◆		

This symbol ◆ indicates the correct answer
